# Supplementary material for: Transcranial focused ultrasound stimulation of cortical and thalamic somatosensory areas in human
Source: PLoS One. 2023 Jul 21;18(7):e0288654. doi: 10.1371/journal.pone.0288654 (PMC10361523; doi:10.1371/journal.pone.0288654)
Supplement: S1 Fig — (DOCX) [file pone.0288654.s001.docx]

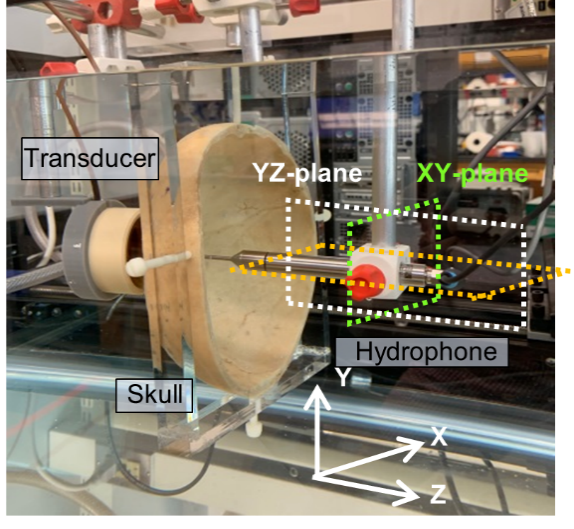


**S1 Fig. An experimental setup for the assessment of the fidelity of acoustic simulation**.

An example of the acoustic field mapping apparatus after transmission through an *ex vivo* human calvarium is shown, with representation of the spatial coordinates and orientation used in the conventions reported in **S1** **Table**. The accuracy of numerical simulation was evaluated using ex vivo adult human skull samples (n = 3, listed ‘SK1’ through ‘SK3’, Brigham and Women’s Hospital IRB Exemption #00000484). First, the samples were imaged with CT using the same method described in human data acquisition, including placement of four fiducial markers over the skull surface. To obtain ‘ground-truth’ information on transcranial propagation of the FUS field inside the skull, the acoustic field was measured in a degassed water tank (oxygen level at 1 ppm as measured using a dissolved oxygen assay kit, K-7512, CHEMetrics, Midland, VA) with and without the skull. Two FUS transducers (D40 and D90) were actuated using the signal-generating circuits and an impedance matching box of the FUS device for human testing. The acoustic field was mapped with 1 mm spatial resolutions covering three different planes (one perpendicular to the sonication direction at the focus and two parallel to the sonication direction). The measurement setup is shown in S1 Fig with conventions used to describe the axis orientation. To simulate the different sonication locations, the skull was moved in five different locations (approximating 5 mm up/down/left/right from the initial location) and the same measurement was taken.
