# Supplementary material for: Transcranial focused ultrasound stimulation of cortical and thalamic somatosensory areas in human
Source: PLoS One. 2023 Jul 21;18(7):e0288654. doi: 10.1371/journal.pone.0288654 (PMC10361523; doi:10.1371/journal.pone.0288654)
Supplement: S2 Fig — (DOCX) [file pone.0288654.s002.docx]

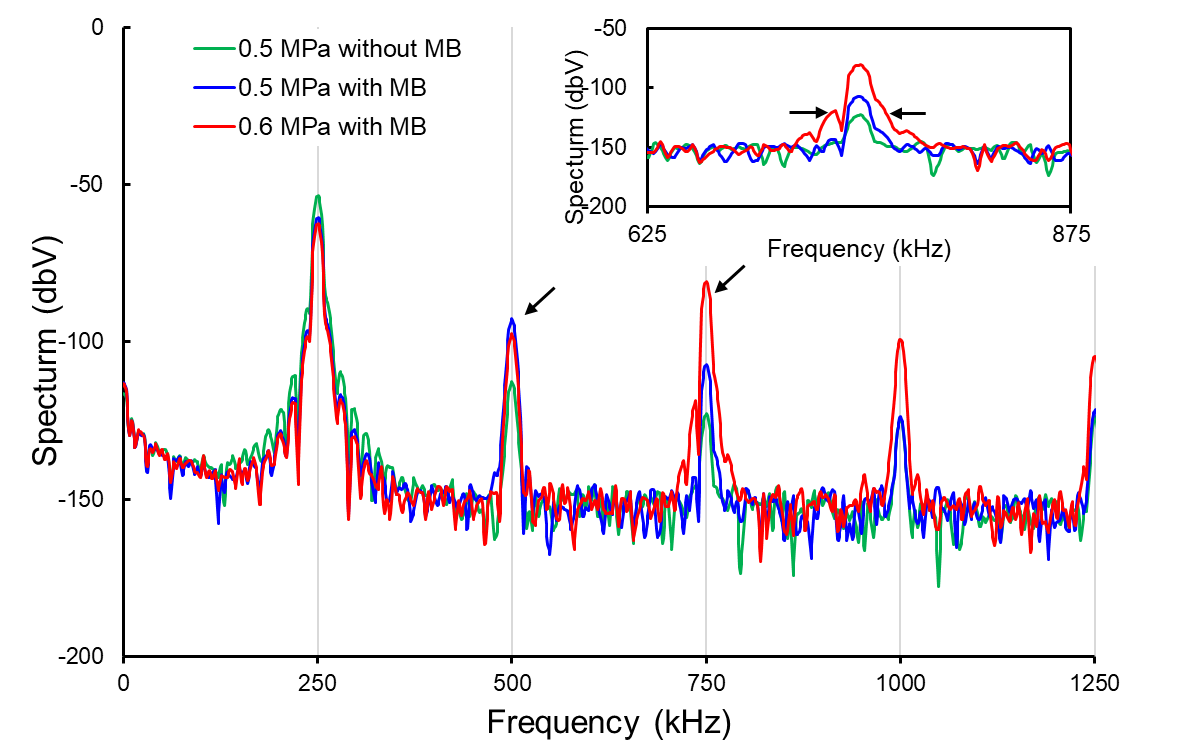


**S2 Fig**. **The spectra of passive cavitation signals measured up to the 5^th^ harmonics (1250 kHz) of FUS sonication.**

The performance of AED was evaluated prior to use in human testing. The use of extremely low acoustic intensity in the current experimental setting, whereby the maximum power output is also hardware-limited for safety, does not induce cavitation. Thus, the device performance was tested by characterizing the changes in amplitude and shape of emission spectra that were associated with cavitation events, which was achieved by sonicating the normal saline bath that contained ultrasound microbubbles (Definity, Lantheus, Billerica, MA 0.1 µL/mL volume concentration). The detector was placed 10 mm away from the acoustic focus and continuous sonication (250 kHz) was applied with a rarefactional pressure of 0.5 MPa (mechanical index of 1.0). The spectra obtained in the absence of microbubbles (MB) are marked by the green line whereby the application of 0.5 MPa rarefactional pressure (in blue line) showed an increase in the spectrum amplitude, particularly at the second and third harmonics (indicated by the arrows). Further increase in pressure (0.6 MPa) increased the spectral amplitude at the third harmonics and started to show broadening of the spectra. The amplitude of frequency spectra at the second and third harmonics (500 and 750 kHz) (blue line, indicated with arrows) visibly increased compared to those obtained without the introduction of microbubbles (green line), indicating the presence of stable cavitation. When the pressure amplitude further increased to 0.6 MPa, the spectrum at the third harmonics started to broaden (red line, also shown in the inset), which indicated the transition to inertial cavitation.
