# Supplementary material for: Transcranial focused ultrasound stimulation of cortical and thalamic somatosensory areas in human
Source: PLoS One. 2023 Jul 21;18(7):e0288654. doi: 10.1371/journal.pone.0288654 (PMC10361523; doi:10.1371/journal.pone.0288654)
Supplement: S3 Fig — (DOCX) [file pone.0288654.s003.docx]

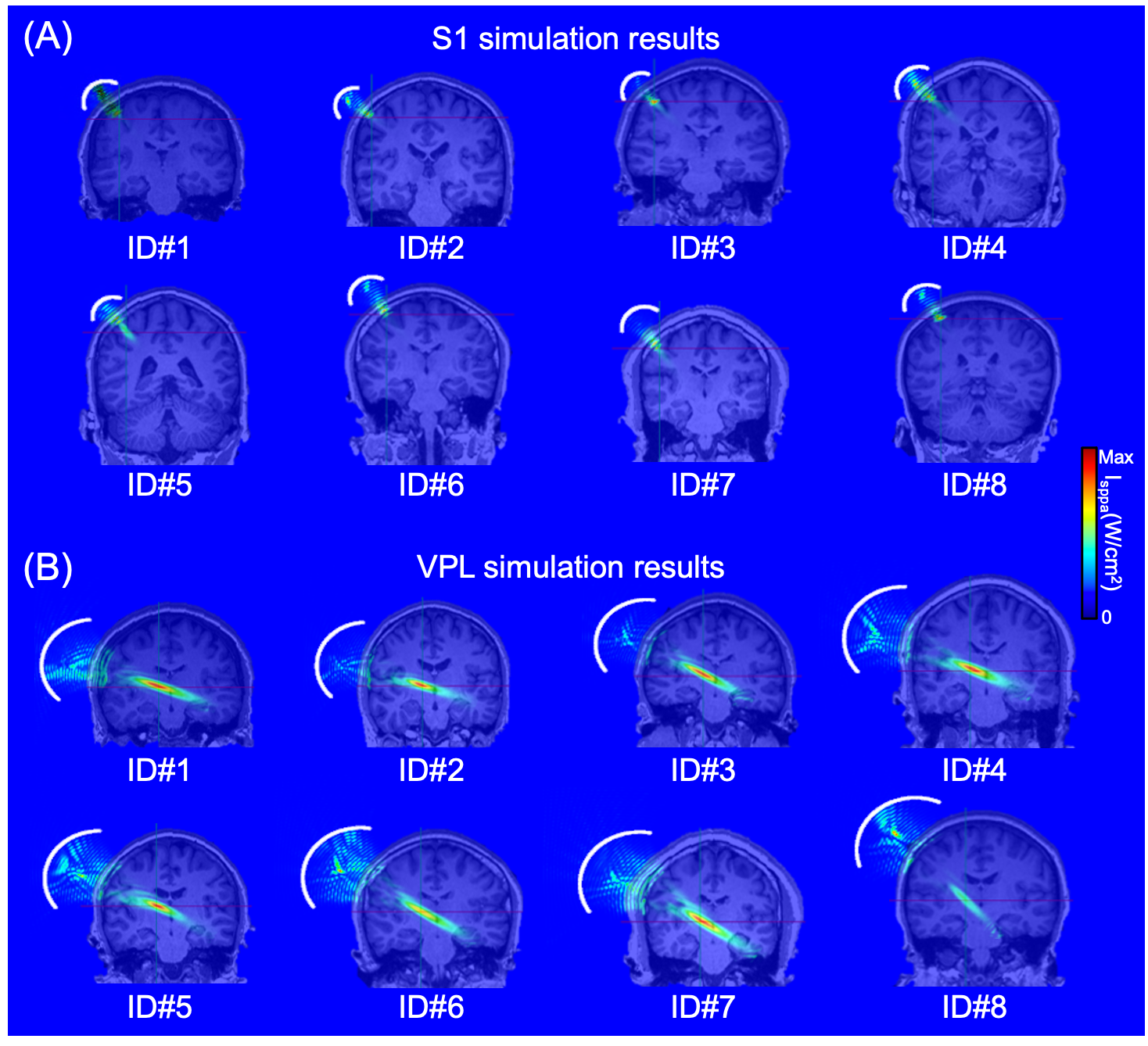


**S3 Fig.** **Spatial profile of acoustic intensity obtained from numerical simulation.** (A) Simulation results from S1 stimulation and (B) from VPL stimulation that were overlaid on the coronal MRI section across eight participants (ID#1 through ID#8) at the maximum intensity. I_sppa_: Spatial-peak pulse-average intensity, S1: Primary somatosensory area, VPL: Ventral posterolateral nucleus.
