# Supplementary material for: Transcranial focused ultrasound stimulation of cortical and thalamic somatosensory areas in human
Source: PLoS One. 2023 Jul 21;18(7):e0288654. doi: 10.1371/journal.pone.0288654 (PMC10361523; doi:10.1371/journal.pone.0288654)
Supplement: S4 Fig — (DOCX) [file pone.0288654.s004.docx]

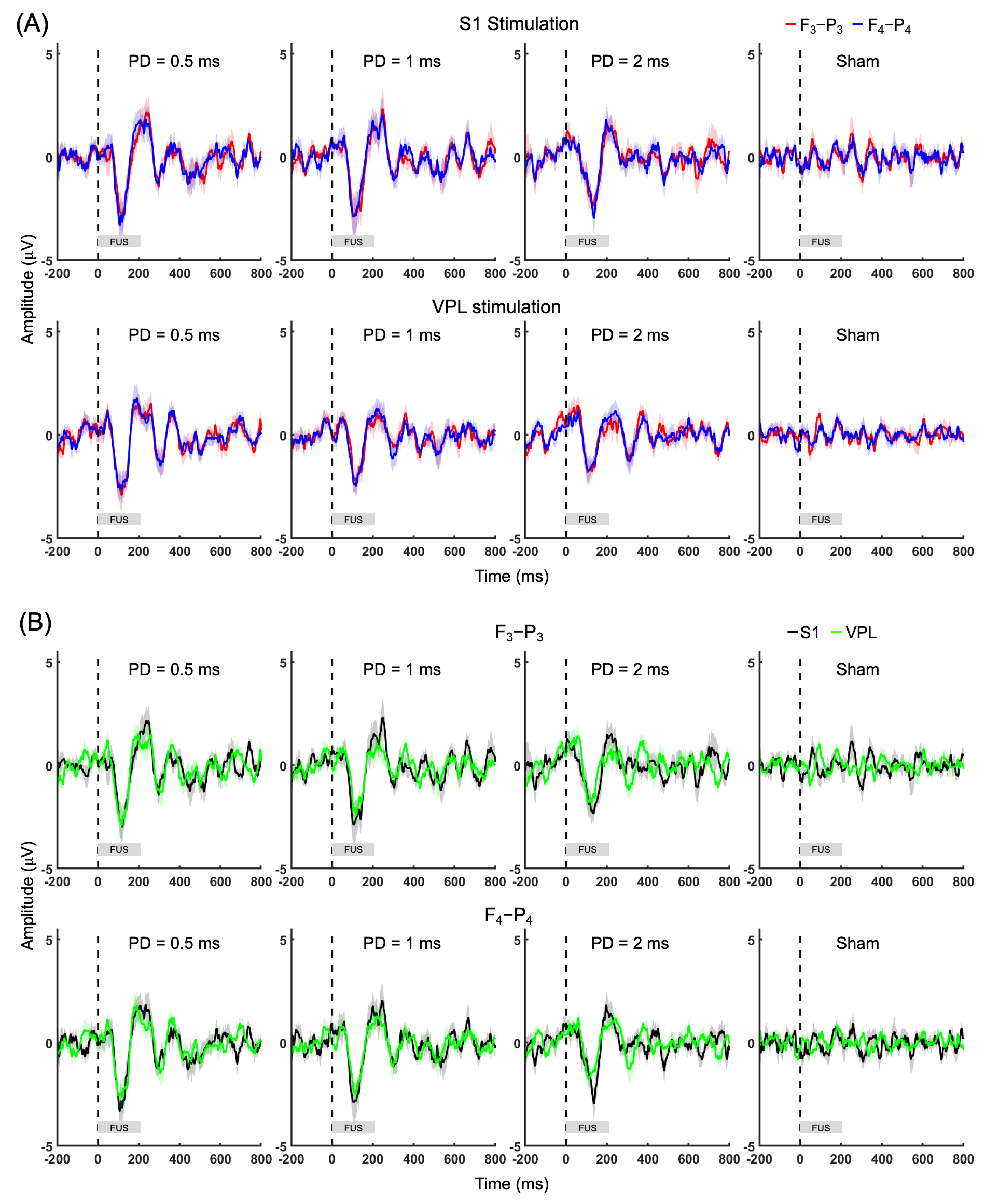


**S4 Fig. Group comparison FEPs between hemispheres and stimulation targets.** (A) Group comparison between the hemispheric FEPs (*i.e.*, F_3_-P_3_ versus F_4_-P_4_) across different PD conditions. (B) Comparison between the stimulation targets (*i.e.*, S1 versus VPL) in different PD conditions. No significant differences in FEPs between the hemispheres (*p* < 0.005) and stimulation targets (*p* < 0.001) were seen. The solid lines and shaded areas indicate group-average FEP and standard errors for all subjects, respectively.
