# Supplementary material for: Transcranial focused ultrasound stimulation of cortical and thalamic somatosensory areas in human
Source: PLoS One. 2023 Jul 21;18(7):e0288654. doi: 10.1371/journal.pone.0288654 (PMC10361523; doi:10.1371/journal.pone.0288654)
Supplement: S5 Fig — (DOCX) [file pone.0288654.s005.docx]

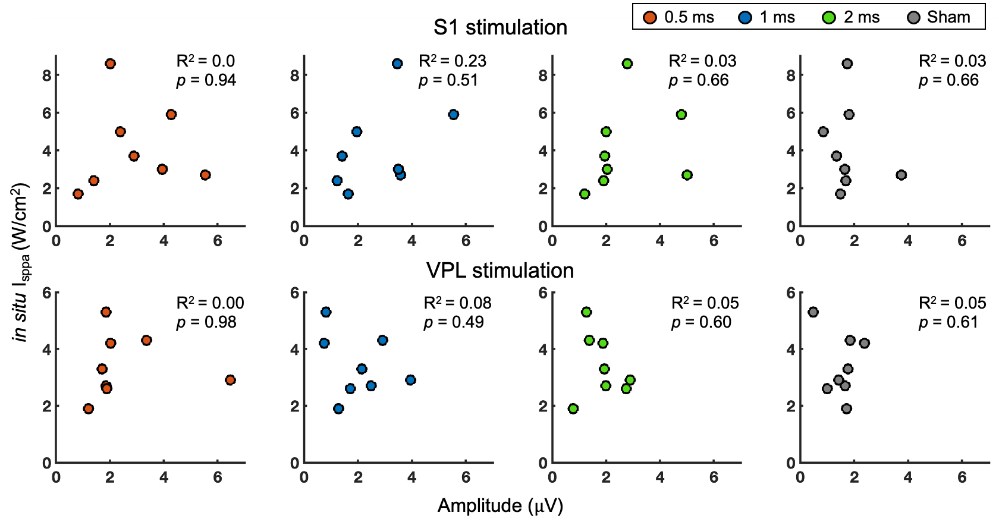


**S5 Fig.** **The comparison between *in situ* I_sppa_ and FEP amplitude across different pulsed durations, including the sham condition.** No correlation was found from stimulation of either the S1 or the VPL. R^2^: linear regression coefficient, *p*: p-value.
