# Supplementary material for: Transcranial focused ultrasound stimulation of cortical and thalamic somatosensory areas in human
Source: PLoS One. 2023 Jul 21;18(7):e0288654. doi: 10.1371/journal.pone.0288654 (PMC10361523; doi:10.1371/journal.pone.0288654)
Supplement: S6 Fig — (DOCX) [file pone.0288654.s006.docx]

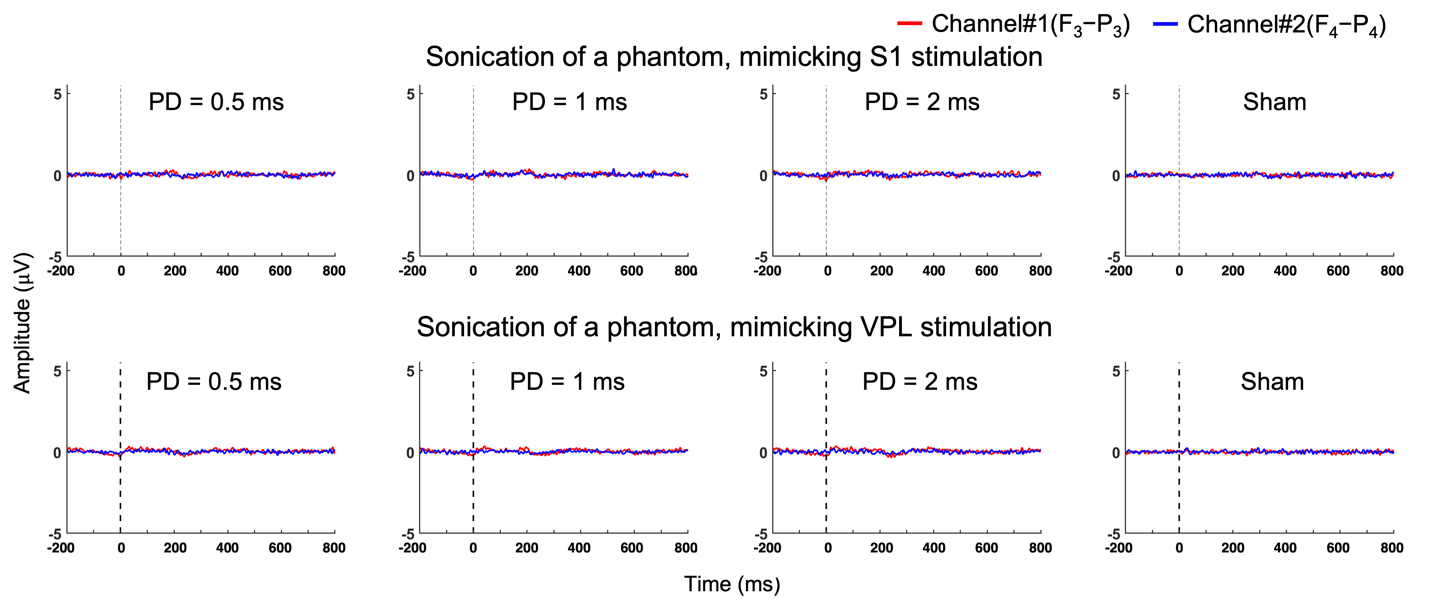


**S6 Fig.** **Examination of sonication-related artifacts in FEP acquisition**. Time-locked acquisition EEG measurement (*i.e.*, F_3_-P_3_ versus F_4_-P_4_) from a gel phantom across different PD conditions which were used in (A) S1 and (B) VPL stimulations. There was no amplitude difference in the EEG time series between the sonication conditions compared to EEG acquired during the sham condition (*i.e.,* without sonication) (*p* < 0.005), suggesting that the observed FEPs were not artifactually affected by the FUS sonication.
