# Supplementary material for: Transcranial focused ultrasound stimulation of cortical and thalamic somatosensory areas in human
Source: PLoS One. 2023 Jul 21;18(7):e0288654. doi: 10.1371/journal.pone.0288654 (PMC10361523; doi:10.1371/journal.pone.0288654)
Supplement: S1 Table — (DOCX) [file pone.0288654.s007.docx]

S1 Table. The difference between the simulation and actual measurement of the FUS focus.

The measured acoustic fields were characterized in terms of focal location (the pressure maximum) and dimensions (length and width of the ellipsoid defined at 90%-maximum) as well as the pressure level at the focus (expressed in % to the one measured without the skull). After co-registering markers that were placed over the transducer and the skull with the corresponding virtual space represented by the CT, numerical simulation was performed to derive the same indices as the acoustic field mapping. Then, the difference between the simulation and actual hydrophone measurement, in terms of focal location (ΔF), dimension of long-axis of focus (ΔL), dimension of short-axis width of focus (ΔW), and pressure ratio (ΔPr), was calculated for each skull sample to assess the simulation accuracy. The measurements taken from the two transducers across three orthogonal planes (i.e., XY, YZ, and XZ planes) are listed. The measurements were taken from three orthogonal planes (*i.e.*, XY, YZ, and XZ). The differences were measured in terms of focal location (ΔF), dimension of long-axis of focus (ΔL), dimension of short-axis width of focus (ΔW), and pressure ratio (ΔPr), across the 3 skull samples (SK1 – SK3) with respect to two FUS transducers (D40 and D90). The value was averaged across five different skull locations (± standard deviation). The maximum of grand mean ΔF, ΔL/W and ΔPr values (across all three planes and transducers) were 1.7 ± 1.0 mm (from D40), 3.0 mm ± 2.5 mm (from D90), and 3.1 ± 2.5% (from D40). All these measures suggest the reliable operation of the simulation algorithm for the measurement setting.

| XY-plane | | | | | |
| --- | --- | --- | --- | --- | --- |
| Transducer | Skull ID | ΔF (mm) | ΔL (mm) | ΔW (mm) | ΔPr (%) |
| D40 | SK1 | 0.9 ± 0.5 | -0.1 ± 0.2 | 0.4 ± 0.7 | -0.9 ± 1.3 |
|  | SK2 | 1.0 ± 0.4 | -1.1 ± 0.4 | -0.4 ± 0.7 | 4.8 ± 2.0 |
|  | SK3 | 0.8 ± 0.1 | -0.6 ± 0.2 | 0.3 ± 0.2 | 2.1 ± 2.4 |
| D90 | SK1 | 1.3 ± 0.4 | 0.1 ± 0.2 | -0.2 ± 0.6 | -5.0 ± 0.4 |
|  | SK2 | 0.9 ± 0.1 | -0.8 ± 0.4 | -0.3 ± 0.4 | 0.0 ± 0.6 |
|  | SK3 | 1.0 ± 0.2 | -0.2 ± 0.2 | -0.4 ± 0.2 | -1.7 ± 0.5 |
| YZ-plane | | | | | |
| D40 | SK1 | 1.8 ± 0.7 | -1.9 ± 1.9 | 0.2 ± 0.4 | -0.2 ± 2.1 |
|  | SK2 | 2.6 ± 1.2 | 1.6 ± 2.6 | -0.2 ± 0.4 | 4.4 ± 2.4 |
|  | SK3 | 1.2 ± 0.2 | -0.6 ± 2.1 | -1.1 ± 0.2 | 1.1 ± 3.9 |
| D90 | SK1 | 2.6 ± 0.6 | -2.5 ± 1.1 | -0.4 ± 0.2 | -5.3 ± 0.3 |
|  | SK2 | 1.4 ± 0.4 | -5.0 ± 2.2 | -0.5 ± 0.4 | -0.8 ± 0.4 |
|  | SK3 | 1.2 ± 0.3 | -4.8 ± 1.1 | -0.5 ± 0.0 | -1.1 ± 0.5 |
| XZ-plane | | | | | |
| D40 | SK1 | 2.7 ± 0.8 | 1.3 ± 2.9 | -0.6 ± 0.8 | -2.7 ± 3.0 |
|  | SK2 | 2.4 ± 1.2 | 1.3 ± 2.4 | -0.9 ± 0.9 | 3.7 ± 1.8 |
|  | SK3 | 1.6 ± 0.3 | 0.7 ± 3.2 | -0.3 ± 0.6 | 2.3 ± 4.3 |
| D90 | SK1 | 1.1 ± 0.7 | -3.2 ± 2.0 | 0.0 ± 0.0 | -7.2 ± 1.6 |
|  | SK2 | 2.3 ± 0.4 | -5.7 ± 1.4 | -0.1 ± 0.5 | -0.5 ± 0.7 |
|  | SK3 | 1.3 ± 0.5 | -4.7 ± 0.8 | -0.3 ± 0.4 | 0.2 ± 0.8 |
