# Supplementary material for: Transcranial focused ultrasound stimulation of cortical and thalamic somatosensory areas in human
Source: PLoS One. 2023 Jul 21;18(7):e0288654. doi: 10.1371/journal.pone.0288654 (PMC10361523; doi:10.1371/journal.pone.0288654)
Supplement: S1 Appendix — (DOCX) [file pone.0288654.s008.docx]

**Supporting information**

**S1 Appendix. *Validation of numerical simulation***

The accuracy of numerical simulation was evaluated using *ex vivo* adult human skull samples (*n* = 3, listed ‘SK1’ through ‘SK3’, Brigham and Women’s Hospital IRB Exemption #00000484). First, the samples were imaged with CT using the same method described in the human data acquisition, including placement of four fiducial markers over the skull surface. To obtain ‘ground-truth’ information on transcranial propagation of the FUS field inside the skull, the acoustic field was measured in a degassed water tank (oxygen level at 1 ppm as measured using a dissolved oxygen assay kit, K-7512, CHEMetrics, Midland, VA) with and without the skull. Two FUS transducers (D40 and D90) were actuated using the signal-generating circuits and an impedance matching box of the FUS device for human testing. The acoustic field was mapped with 1 mm spatial resolutions covering three different planes (one perpendicular to the sonication direction at the focus and two parallel to the sonication direction). The measurement setup is shown in S1 Fig with conventions used to describe the axis orientation. To simulate the different sonication locations, the skull was moved in five different locations (approximating 5 mm up/down/left/right from the initial location) and the same measurement was taken.

The measured acoustic fields were characterized in terms of focal location (the pressure maximum) and dimensions (length and width of the ellipsoid defined at 90%-maximum) as well as the pressure level at the focus (expressed in % to the one measured without the skull). After co-registering markers that were placed over the transducer and the skull with the corresponding virtual space represented by the CT, numerical simulation was performed to derive the same indices as the acoustic field mapping. Then, the difference between the simulation and actual hydrophone measurement, in terms of focal location (ΔF), dimension of long-axis of focus (ΔL), dimension of short-axis width of focus (ΔW), and pressure ratio (ΔPr), was calculated for each skull sample to assess the simulation accuracy. The measurements taken from the two transducers across three orthogonal planes (i.e., XY, YZ, and XZ planes) are listed in S1 Table. The maximum of grand mean ΔF, ΔL/W and ΔPr values (across all three planes and transducers) were 1.7 ± 1.0 mm (from D40), 3.0 mm ± 2.5 mm (from D90), and 3.1 ± 2.5% (from D40). All these measures suggest the reliable operation of the simulation algorithm for the measurement setting.
