# Supplementary material for: Transcranial focused ultrasound stimulation of cortical and thalamic somatosensory areas in human
Source: PLoS One. 2023 Jul 21;18(7):e0288654. doi: 10.1371/journal.pone.0288654 (PMC10361523; doi:10.1371/journal.pone.0288654)
Supplement: Attachment — Submitted filename: Response to reviewers.pdf [file pone.0288654.s009.pdf]

Response to Review
Editorial Office – PLOS ONE

RE: Revision of a manuscript (ID: PONE-D-23-00728) entitled, 'Transcranial focused ultrasound stimulation of cortical and thalamic somatosensory areas in human'.

We would like to thank for granting us the opportunity to revise our submission. The Reviewers' comments were very helpful toward improving the manuscript. We have addressed each point raised by the Reviewers and have listed the responses in blue text below, along with highlighted changes in the manuscript. We assigned numbers to the comments in sequence.

To address the editor's concern over the availability of data, all data files (listed below) were uploaded to shared data repository https://dataverse.harvard.edu/dataset.xhtml?persistentId=doi:10.7910/DVN/380AEI for access, with a metafile indicating the data curation.

- Median nerve stimulation SSEP data (Fig. 3A)
- FEP data (Fig. 3B)
- AEP data (Fig. 4)
- rsfMRI data (Fig. 5 and 6)

Reviewer's comment:

Please use the space provided to explain your answers to the questions above. You may also include additional comments for the author, including concerns about dual publication, research ethics, or publication ethics. (Please upload your review as an attachment if it exceeds 20,000 characters)

Reviewer #1: This study examines the online and offline effects of focused ultrasound stimulation of the somatosensory cortex and VPL nucleus of the thalamus, in humans. The use of a specially designed headgear for transducer positioning, individual simulations based on CT scans, real-time image guidance and testing of auditory evoked potentials (AEPs) - albeit in a separate participant group - are some strong points of this paper. However, at this point, the reported physiological effects are not convincing, and I have some major concerns about the study.
We appreciate the Reviewer for the overall positive evaluation. We listed our responses below, including our responses to reviewer's concern over the physiological effects from the FUS stimulation,

My main concern is regarding the timeline of measurement of the offline effects.
1. The baseline resting state functional connectivity (rsFC) measurement is made several days before the post FUS measurements. Can you provide any evidence that rsFC measurements are stable and reliable over such long periods? This is particularly important given the small sample size (n = 8).

(Response) We did not conduct a separate study that examined the inter-session reproducibility of rsFC between the initial and the second connectivity measurement following FUS stimulation. Based on a study by Anderson and colleagues (reference [86]) that examined the variability of FCs from a subject over three-week period (measured five times), the FC changes that occurred within a week did not exceed the range of normal variability. Another study (reference [87]) that examined the resting state networks (RSNs, e.g., default mode, attention, executive-function, and salient networks) weekly for 3.5 ys from a healthy individual also found that a week period does not introduce more variability beyond either intersession or the inter-subject variability (a degree of linear trend and annual periodicity were observed depending on the types of RSNs). Although these studies suggest that the time gap in the present study between Visit #1 and #3 was unlikely to introduce a significant variability that affected the overall interpretation of the results,

we acknowledged the limitations associated with the study design and small sample size in the Discussion section.

(Line 737-743)
"We acknowledge that the present study lacked separate assessment of the degree of normal variabilities in rsFC across the study period, without administering FUS stimulation. Although previous studies have shown that a week time gap does not introduce more variability to rsFC beyond normal inter-session variability across much longer time period (e.g., three weeks [86] or 3.5 year [87]), the time gap between the Visit#1 and #3 (about a week) might have contributed individual fluctuations in the rsFC. Acquisition of baseline rsFC data right before the initial FUS stimulation session may help reduce this potential confounder."…

[86] Anderson JS, Ferguson MA, Lopez-Larson M, Yurgelun-Todd D. Reproducibility of single-subject functional connectivity measurements. AJNR Am J Neuroradiol. 2011;32(3):548-55.
[87] Choe AS, Jones CK, Joel SE, Muschelli J, Belegu V, Caffo BS, et al. Reproducibility and temporal structure in weekly resting-state fMRI over a period of 3.5 years. PloS one. 2015;10(10):e0140134.

2. What is the rationale for making the post FUS rsFC measurement 1 hr after VPL sonication, but 1 week after S1 sonication. In fact, on line 670, the authors mention that any FUS effects may be washed out after a week. This makes any reported FUS effects on S1 questionable. Also, why was the sonication of S1 and VPL not counter-balanced?
(Response) A one-week time gap between two FUS stimulation sessions was introduced to allow for monitoring any unexpected safety responses from the subjects while participants may consider retracting from the study. Inclusion of both S1 and VPL in a single session was avoided due to expected fatigue from a stimulation session, which will last far more than an hour (including the needs for a separate image-guidance). The counterbalanced randomization or cross-over design between the S1 and VPL stimulation sessions would further confound the interpretation of the rsFC data among small sample size at this exploratory stage, which forced us to homogenize the stimulation session sequence. We are grateful for the comment, and Methods section was revised to provide the rationale. The study limitations were also revisited in the Discussion section.

(Line 143-147)
… "A time gap of one week between two FUS stimulation sessions was introduced to allow for the observation of any unexpected safety-related side effects from participants while allowing them enough time to consider retraction from the study. Stimulation of both S1 and VPL within a single session was not conducted to avoid fatigue from long experimental time, which would take far more than an hour (~ 2 hrs) involving the needs for separate image-guidance procedures."…

(Line 743-754)
…"We also note that the observed changes in FC in Visit #3 may not be solely related to the VPL stimulation and may include effects from the S1 stimulation (which was given about a week prior). Zhang and colleagues suggested that 7 days may be considered a sufficient washout period after stimulating the primary hand motor area using rTUS [11]; however, it is possible that modulatory effects of the S1 may remain about a week later, being mixed with the effects from thalamic stimulation. Counter-balanced randomization or a crossover between the S1 and VPL stimulation sessions can be sought after; however, considering the small number of subjects in the study, it may introduce potential mutual interactions between the stimulatory effects, which serve as further confounds during the interpretation of the data. In addition, contributions from different PD conditions on rsFC were not examined as subjects underwent the FUS session containing all three PD conditions mixed in a randomized fashion. These limitations call for separate rsFC data acquisitions per stimulation targets, as well as per sonication parameter to achieve more robust rsFC analysis to reveal the therapeutic utility of transcranial FUS."

3. Line 25 – FUS induced or evoked? This is repeated several times in the paper. In EEG literature, "induced" typically refers to a non-phase-locked change in e.g., oscillatory brain activity, whereas "evoked" refers to phase-locked responses (such as classical ERPs)

(Response) We thank the reviewer for the comment. The expression was changed to "evoked" to refer to the phase-locked responses.

(Line 29)
In abstract, …"These FUS-evoked EEG potentials (FEP) were observed from both brain hemispheres and shared similarities with somatosensory evoked potentials (SSEP) from median nerve stimulation."…
(Line 78)
… "In anesthetized rats, FUS stimulation of the somatosensory areas yielded different features in EEG somatosensory evoked potential (SSEP; evoked by the unilateral electrical stimulation of the hind limb), which lasted over 35 min after sonication [25]."…
(Line 102)
… "The first segment evaluated FUS-evoked EEG potentials (referred to as 'FEP' herein)"…
(Line 135)
Fig. 1. Experimental procedure. Acronyms used — CT: Computed tomography, aMRI: Anatomical magnetic resonance imaging, fMRI: Functional magnetic resonance imaging, rs-fMRI: Resting-state functional magnetic resonance imaging, NE: Neurological evaluation, SSEP: Somatosensory evoked potentials evoked by median nerve (m.n.) stimulation,"…
(Line 465-466)
… "FUS elicited distinct signal features that shared similarities to the SSEP evoked by median nerve stimulation (e.g., negative peaks of 37–67 ms latency; Fig. 3A)."…

4. Line 73 – offline effects are still transient. They are not permanent.

(Response) The sentence was deleted accordingly.

5. The fMRI images have relatively low resolution with gaps – how does this affect the connectivity estimates?

(Response) We thank the reviewer for the comment and agreed that the slice gap used in the present study (*i.e.,* 1 mm) was rather substantial, even considering the technique to reduce potential contributions from the crosstalk between the slice selection. Higher spatial resolution on the order of 2.5 mm^3 isovoxel size would benefit the interpretation of the FC, and we acknowledged the limitations in the Discussion section about the importance of imaging parameters in rsfMRI data acquisition, with the inclusion of a reference on multiband multi-echo fMRI acquisition that can be adopted to improve sensitivity and reproducibility of the rsFC (Cohen et al., 2021).

(Line 778-781)
… "We also note that the 1-mm slice gap used in rsfMRI data acquisition was rather wide even considering the potential contributions from the signal crosstalk between the slice selection of MRI. Higher spatial resolution, on the order of 2.5 mm^3 isovoxel size, and adoption of an imaging sequence such as multiband multi-echo fMRI [94], would improve sensitivity and reproducibility of the rsFC."…

[94] Cohen AD, Yang B, Fernandez B, Banerjee S, Wang Y. Improved resting state functional connectivity sensitivity and reproducibility using a multiband multi-echo acquisition. NeuroImage. 2021;225:117461.

6. Please explain the cavitation detection in further detail – does the described method detect actual occurrence of stable vs inertial cavitation or assesses the probability of cavitation? Does it provide any information about potential location of cavitation? Is there in vitro evidence that this particular setup is able to pick up stable/inertial cavitation?

(Response) The use of a single detector, implemented in the present study, does not provide information on the potential location of the cavitation which is possible by using multi-array detectors (Gray et al. 2021 [46]). Due to the use of an extremely low intensity, which does not induce cavitation in the current setting, the device performance was tested through examination of the frequency spectrum by applying sonication to the normal saline containing 0.1 mL volume concentration of microbubble-based ultrasound contrast agent. The Methods section was revised with inclusion of cavitation measurement in Supporting Information. We also included the technique (reference [46]), which can be adopted to locate the potential area of cavitation.

(Line 314-319)

… "Although a multi-array detector configuration may provide information on the potential location of cavitation [46], we adopted a single detector element for its small size that can be placed between the EEG electrodes. The device performance was evaluated by detecting the intentional cavitation events by sonicating microbubbles (Definity, Lantheus, Billerica, MA; prepared in 0.1 mL volume concentration in normal saline) (**Supporting Information, S2 Appendix**)."

S2 Appendix. Performance validation of passive cavitation detection

The performance of AED was evaluated prior to use in human testing. The use of extremely low acoustic intensity in the current experimental setting, whereby the maximum power output is also hardware-limited for safety, does not induce cavitation. Thus, the device performance was tested by characterizing the changes in amplitude and shape of emission spectra that were associated with cavitation events, which was achieved by sonicating the normal saline bath that contained ultrasound microbubbles (Definity, Lantheus, Billerica, MA 0.1 µL/mL volume concentration). The detector was placed 10 mm away from the acoustic focus and continuous sonication (250 kHz) was applied with a rarefactional pressure of 0.5 MPa (mechanical index of 1.0). The amplitude of frequency spectra at the second and third harmonics (500 and 750 kHz) (blue line, **S2 Fig**, indicated with arrows) visibly increased compared to those obtained without the introduction of microbubbles (**S2 Fig**, green line), indicating the presence of stable cavitation. When the pressure amplitude further increased to 0.6 MPa, the spectrum at the third harmonics started to broaden (**S2 Fig**, red line, also shown in the inset), which indicated the transition to inertial cavitation.

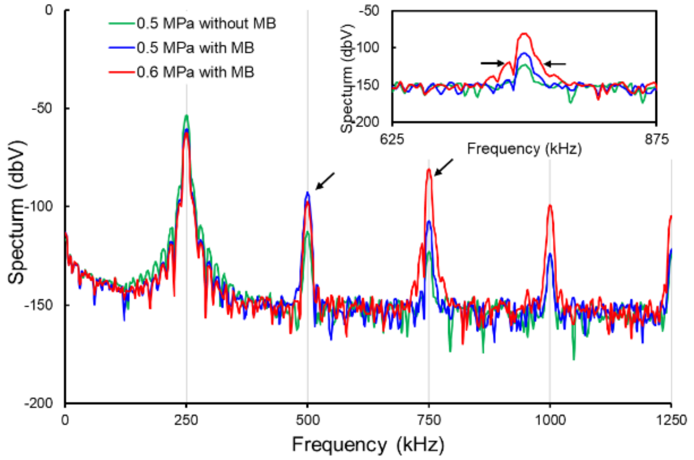

S2 Fig. The spectra of passive cavitation signals measured up to the 5th harmonics (1250 kHz) of FUS sonication. The spectra obtained in the absence of microbubbles (MB) are marked by the green line whereby the application of 0.5 MPa rarefactional pressure (in blue line) showed an increase in the spectrum

amplitude, particularly at the second and third harmonics (indicated by the arrows). Further increase in pressure (0.6 MPa) increased the spectral amplitude at the third harmonics and started to show broadening of the spectra.

[46] Gray MD, Elbes D, Paverd C, Lyka E, Coviello CM, Cleveland RO, et al. Dual-array passive acoustic mapping for cavitation imaging with enhanced 2-D resolution. IEEE Trans Ultrason Ferroelectr Freq Control. 2020;68(3):647-63.

7. Line 553 – what do you mean by 'perception toward brain stimulation'?

(Response) The texts were revised to 'any sensation perceived during the brain stimulation'.

(Line 612-615)
… "In addition, as sensations perceived during brain stimulation may vary widely in time as well as between individuals (as also observed in case of TMS stimulation [58, 59]), further investigation is needed to evaluate intra- and inter-session reproducibility of FUS brain stimulation and associated perception of sensations, based on the use of a fixed set of sonication parameters."

8. Line 566 – how did you confirm that the EEG did not contain stimulation-related artifacts?

(Response) We acquired EEG data using gel phantom (updated the Methods section) and reported the findings in the **Supporting information S6 Fig.**

(Line 334-342)
Assessment of presence of EEG signal artifact due to sonication
To assess the presence of sonication-related artifacts in the recorded FEP, we measured EEG signals from a hydrogel phantom simultaneously with application of FUS. A hydrogel phantom was prepared by dissolving PVA in 150 mL of distilled water (7% weight/volume concentration), which was molded in a 14 cm diameter petri dish and subjected to 2 freeze-thaw cycles to make it in the form of a 10 mm-thick disk. After placing the gel phantom over a 7 mm-thick rubber pad for acoustic absorption, the transducer was positioned over the surface of the phantom along with acoustic coupling gel. Then, EEG electrodes were placed around the transducer mimicking the location used in vivo experiment. The same experimental procedures as the in vivo experiment were used to separately acquire the EEG data from two transducers.

(Line 487-489)
… "From the assessment of potential sonication-related artifacts in EEG measurement, we did not find differences in EEG time-series amplitudes between the sonication conditions and the sham (p < 0.005; **Supporting information S6 Fig.**)."

(Line 627-630)
… "We also note that FEP, unlike electrical/magnetic stimulation of the brain, did not contain any significant stimulation-related artifacts (**Supporting information S6 Fig.**), which indicates that acoustic stimulation is compatible with studies where minimal EEG artifacts are desired."

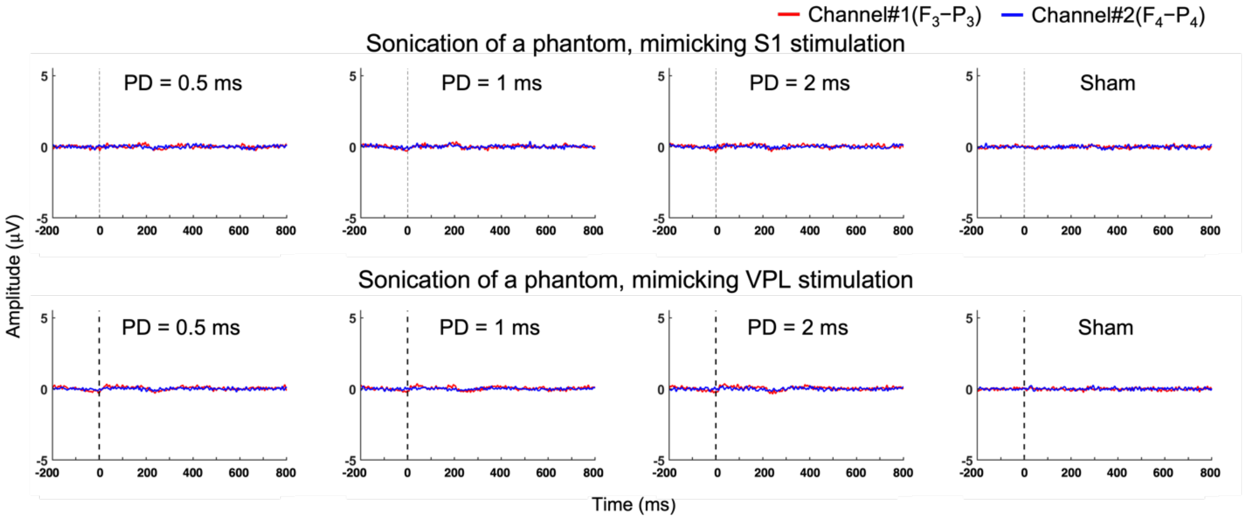

S6 Fig. Examination of sonication-related artifacts in FEP acquisition. Time-locked acquisition EEG measurement (*i.e.*, F_3-P_3 versus F_4-P_4) from a gel phantom across different PD conditions which were used in (A) S1 and (B) VPL stimulations. There was no amplitude difference in the EEG time series between the sonication conditions compared to EEG acquired during the sham condition (*i.e.,* without sonication) ($p < 0.005$), suggesting that the observed FEPs were not artifactually affected by the FUS sonication.

9. While it is helpful to show that sound alone did not evoke AEPs in the used electrodes, the US is largely bone conducted and may have a different quality. Therefore, it is not possible to entirely rule out auditory confounds, even if the sounds used for eliciting AEPs are loud enough. This should be mentioned as a limitation.

(Response) The limitation was added in the Discussion section. We thank the reviewer for the comment.

(Line 685-689)
… "As the characteristics of sound conduction through the bone may differ from the one used in measuring the AEP, it is not possible to entirely rule out auditory confounds during stimulation. To remedy the auditory confounder, auditory masking [64] (*e.g.,* presentation of audio tones synchronized with sonication or scanner noise from MRI) or amplitude modulation of acoustic waves [68] can be sought after."

10. For participants who experienced a tactile/vibratory sensation on the scalp – the FEP may in fact be a sensory evoked potential. Please mention this as a limitation.
(Response) The limitation was added in the Discussion section.

(Line 668-673)
… "The SSEP from unilateral median nerve stimulation showed bi-hemispheric EEG peaks (Fig. 3A), albeit at a reduced amplitude from the opposite hemisphere, which also partially supports our postulation. However, we note that the measured FEP may also reflect sensory evoked potential to the tactile sensation on the scalp, although only one participant experienced such a sensation (ID#7 under VPL stimulation). Thus, further investigation is needed to isolate the cause for the observed bilateral FEP."

Reviewer #2: In this study, Kim et al. investigate the effects of transcranial focused ultrasound stimulation (FUS) on the thalamus (ventral posterolateral nucleus) and the somatosensory cortex. They evaluate ultrasound-evoked potentials and offline effects of FUS on resting state functional connectivity. Auditory evoked potentials by a tone are compared to FUS evoked potentials to rule out the possibility that FUS-mediated effects might be confounded by stimulating the auditory system. This is an interesting report, well written and easy to follow. I have only a few questions and suggestions that the authors may want to consider:

We appreciate the Reviewer for the overall positive evaluation.

1.The paper is limited by its small sample size, which calls into question the generalizability of the results. I suggest the author clearly mention this limitation in the discussion. How large are the effect sizes in MRI resting data results? I am not asking the authors to scan more participants for the current study, but estimation of power and effects size would help other researchers who plan to run similar experiments in future.

(Response) We thank the reviewer for pointing out the limitation associated with rather small sample size in the present study. We added group-averaged fisher's z transformed correlation coefficient, *r* from the brain regions in revised Table 2 to represent the effect sizes of rs-FC from each visit. The ranges of the effect sizes from Visit #3 were 0.22–0.85 whereas the ranges from Visit #1 and Visit #5 were 0.03–0.44 and -0.04–0.36, respectively. Conclusive derivation of the effects size and power analysis ramifying from the FUS stimulation on human neural circuits is difficult to ascertain from a small sample size, especially regarding the complex cognitive or mental phenotypic differences. As suggested, the limitations of the current study were discussed, along with improvement in rsfMRI data acquisition (as also commented from #5 by Reviewer#1)

(Line 800-805)

… "The generalization of our findings on rsFC was limited by the small sample size (n = 8), rendering conclusive derivation of the effects size and power analysis difficult to ascertain. Although the central tendencies of functional brain organization among different experimental groups can be captured from a group of ~24 subjects [97, 98], lack of information regarding normal inter-subject/inter-session variabilities of rsFC data in the context of FUS-mediated brain warrants further investigations involving a greater number of study participants. As FUS stimulation may accompany auditory perception that can impact the interpretation of neural responses, further remedial countermeasures are also urgently needed."

Table 2. Brain regions showing greater rsFC in Visit #3 compared to Visits #1 and #5 (*i.e.,* Visit #3 > Visit #1 and Visit #3 > Visit #5) with respect to the sonicated seed areas, arranged in a descending order in terms of maximum *t*-value within the cluster (Bonferroni corrected *p* < 0.05 with a cluster size of a minimum 30 connected voxels). The averaged rsFC levels (*i.e.,* fisher's z transformed correlation coefficient, *r*) at the brain regions across subjects were represented as the effect size. No significant change in rsFC patterns was observed comparing Visit # 1 > Visit #3 and Visit #5 > Visit #3.

Visit #3 > Visit #1						
Seed area	*AAL/BA*	*Size*	*Foci in mm* (*x*, *y*, *z*)	*Peak intensity* (*t-value*)	*Effect size (r)* Visit#3 (SE)	Visit#1 (SE)
S1 (R)	PrCG, PoCG, PCL (L), MCC (m) / BA 4, 6, 24	326	-21, -19, 76	5.25	0.52 (0.15)	0.12 (0.12)
	SMG (L) / BA 40	32	-57, -52, 34	4.41	0.15 (0.09)	-0.15 (0.06)
	SMG, IPL (R) / BA 40	102	54, -40, 46	4.32	0.30	-0.01

					(0.10)	(0.11)
VPL (R)	Insula, Putamen, FG (R)	230	33, -22, 4	5.98	0.72 (0.19)	0.44 (0.22)
	MTG, ITG (L) / BA 21	46	-54, -22, -20	5.73	0.26 (0.16)	0.00 (0.12)
	PrCG, SFG, SMA (R) / BA 4, 6	210	18, -13, 67	5.40	0.34 (0.11)	0.09 (0.16)
	SFG (L), SFG (m) / BA 10	159	-18, 62, 10	5.38	0.22 (0.15)	-0.12 (0.15)
	STG, MTG (R) / BA 22	116	51, -7, 1	5.03	0.46 (0.23)	0.20 (0.16)
	IFG (L) / BA 45	120	-51, 35, -2	4.92	0.27 (0.15)	0.03 (0.14)
	MCC (m) / BA 24	79	9, -10, 40	4.66	0.46 (0.17)	0.24 (0.11)
	SMA (L) / BA 6	74	-3, 8, 70	4.14	0.29 (0.18)	0.03 (0.16)
Visit #3 > Visit #5						
					Visit#3	Visit#5
S1 (R)	PrCG, PoCG (L), PCL (L) / BA 4	114	-30, -28, 67	4.47	0.85 (0.11)	0.36 (0.07)
	Calcarine (L)	30	-3, -64, 4	4.01	0.24 (0.08)	-0.04 (0.08)
VPL (R)	PrCG (R) / BA 4	62	21, -25, 52	5.62	0.46 (0.19)	0.13 (0.12)
	Insular, Putamen (R)	83	33, -22, 4	4.48	0.72 (0.19)	0.52 (0.19)
	MCC (m) / BA 24, 32	64	-3, 14, 34	4.05	0.48 (0.25)	0.17 (0.17)
Visit #1 > Visit #3 & Visit #5 > Visit #3						
S1 (R)	None detected					
VPL (R)						

AAL: Automated anatomical labeling, BA: Brodmann area, SE: standard error, L: Left, R: Right, m: Medial, S1: Primary somatosensory area, VPL: Ventral posterolateral nucleus, PrCG: Precentral gyrus, PoCG: Postcentral gyrus, PCL: Paracentral lobule, SMG: Supramarginal gyrus, IPL: Inferior parietal lobule, MCC: Middle cingulate cortex, MTG: Middle temporal gyrus, ITG: Inferior temporal gyrus, SFG: Superior frontal gyrus, SMA: Supplementary motor area, STG: Superior temporal gyrus, IFG: Inferior frontal gyrus, IFG: Inferior frontal gyrus.

(Line 778-781)

… "We also note that the 1-mm slice gap used in rsfMRI data acquisition was rather wide even considering the potential contributions from the signal crosstalk between the slice selection of MRI. Higher spatial resolution, on the order of 2.5 mm^3 isovoxel size, and adoption of an imaging sequence such as multiband multi-echo fMRI [94], would improve sensitivity and reproducibility of the rsFC."…

[94] Cohen AD, Yang B, Fernandez B, Banerjee S, Wang Y. Improved resting state functional connectivity sensitivity and reproducibility using a multiband multi-echo acquisition. NeuroImage. 2021;225:117461

[97] Szucs D, Ioannidis JP. Sample size evolution in neuroimaging research: An evaluation of highly-cited studies (1990–2012) and of latest practices (2017–2018) in high-impact journals. NeuroImage. 2020;221:117164.

[98] Marek S, Tervo-Clemmens B, Calabro FJ, Montez DF, Kay BP, Hatoum AS, et al. Reproducible brain-wide association studies require thousands of individuals. Nature. 2022;603(7902):654-60.

2. The study could have benefited greatly from having an active sham control. Please add this limitation to the discussion.

(Response) We revised the Discussion section to address the limitation.

(Line 783-788)
… "Instead of the passive sham condition used in the present study, addition of an active sham control condition, for example, a simple inversion of a FUS transducer facing toward outside of the brain [6, 10], may benefit studies in humans. Advanced active sham control can be sought after by sonicating the brain regions away from the targeted regions [28]; however, the selected target locations to create such a sham condition are limited in the presence of highly extensive functional connectivity in the human brain and would warrant further investigation."…

3. Please provide more information on how spatial errors due to mislocalization of targets were calculated. How robust are these measurements in individual participants? How computationally efficient and time consuming are the numerical calculations? Please add this information to the method section.

(Response) Both on-site and retrospective numerical evaluation of the acoustic propagation was conducted using the same computational platform. The implementation and computational time were described in the Methods section. The accuracy of the simulation algorithm was evaluated using *ex vivo* adult human skull samples and shown in **Supporting information**.

(Line 288-295)
… "The accuracy of the simulation algorithm was evaluated using ex vivo adult human skull samples (n = 3; see **S1 Appendix**. "**Validation of numerical simulation**" section for details, and **Supporting information S1 Fig**), and the averaged error in estimating the acoustic focal location/dimension (< 5 mm) and in situ pressure (< 10%) suggested its reliable operation. The use of GPU-based parallel computation enabled semi real-time (less than 40 s) feedback to the operator. If the location of the focus estimated through simulation deviated greater than 4 mm from the intended target, the transducer was repositioned. After locking the transducer location in place with respect to the headgear, the FUS session commenced."

(Line 297-304)
Retrospective numerical evaluation of acoustic propagation
Despite the use of image-guidance and simulation-assisted initial placement of the transducer, imperfection of the manual placement of the transducer and the acoustic propagation through the skull may introduce unintended spatial error and additional intensity attenuation. Thus, we acquired the sonication geometry upon completion of each stimulation session and re-evaluated the acoustic propagation profile within individuals' calvarium through the simulation using the same simulation platform deployed for image-guidance. The spatial error between the geometric target coordinates and the estimated maxima of the acoustic focus in the brain was calculated.

4. I am wondering whether the targets, especially the thalamus, might be underdosed? What was the rationale for choosing the intensities (14.7 and 9.1 for S1 and thalamus respectively, in free water). Please address this issue in the discussion.

(Response) We are grateful for this important insight. Although the free-water (unattenuated) intensity was lower at the thalamic target than the S1, the range of estimated *in situ* intensity at the thalamic target was maintained relatively similar to the one used in the S1 stimulation, except for one subject (ID#7, 8.6W/cm^2 **Table 1**). Since we cannot completely exclude the possibility of underdosing the VPL stimulation, we addressed this issue in the Discussion section, acknowledging the needs for equal dosing. We also included the needs for more rapid numerical simulation techniques.

(Line 437-441)
… "We note that exceptionally high intensity (8.6 W/cm^2) from one subject (ID7; **Table 1**) contributed to greater group-averaged *in situ* I_{sppa} in case of S1 stimulation than the thalamic stimulation. When the values from ID7 were excluded, the *in situ* I_{sppa} was not different between stimulation of S1 and VPL (3.5 ± 1.5 and 3.5 ± 1.2 W/cm^2, respectively, paired *t*-test, $p = 0.96$)."

(Line 590-597)
… "In addition, we note that the use of a fixed incident I_{sppa} in the present study yielded individually different in situ intensities at the focus, potentially underdosing the thalamic target in one of participants (ID#7, Table 1). To maintain the same *in situ* intensity across all participants, a more rapid, real-time numerical simulation algorithm such as deep-learning-based network or a differential revolution [54, 55] can be used to estimate the derating factor specific to an individual and sonication geometry. Commercial robotic positioning devices, which are available to apply TMS coils in the desired orientation and location [56], may also be conjunctionally used for reproducible FUS stimulation."

5. It is interesting that the participants subjectively reported auditory or somatosensory experiences. I am wondering whether the participants were instructed to expect such sensations or not? Did any participant report those sensations during the sham condition?

(Response) The participants were not informed regarding the presence, including types, of any sensations prior to the sonication. Any motion (including verbal communications unless needed for safety reason) was discouraged. As the verum and sham conditions were randomly presented (computer-generated) within the sonication session, we were not able to obtain information on experience per different conditions. The Methods section was revised, along with the discussion describing the needs for separate evaluations per each experimental condition.

(Line 150-152)
… "The participants were not informed regarding the presence, including types, of any sensation prior to the sonication while any verbal descriptors that may bias the perception were not used by the study staff throughout the study."

(Line 612-615)
… "In addition, as sensations perceived during brain stimulation may vary widely in time as well as between individuals (as also observed in case of TMS stimulation [58, 59]), further investigation is needed to evaluate intra- and inter-session reproducibility of FUS brain stimulation and associated perception of sensations, based on the use of a fixed set of sonication parameters."

6. Maybe I have missed it, but although the SSEPs and FEPs look very similar, at least by eyes, it would be more appropriate to directly test whether they are statistically different or not. Furthermore, I am not entirely sure whether one can conclude that since SSEPs and FEPs are similar, FUS has activated a common pathway … (Line 565). This is likely but not a fact though. I suggest to tone down this statement.

(Response) As SSEP and FEP originates from different neural responses (SSEP reflects afferent brain activity responding to peripheral stimulation whereas FEP detects the neuronal activity caused by direct brain stimulation), we believe that that direct statistical comparisons were not meaningful. We noted this difference in more detail, in terms of the timing of first negative and positive peaks and their magnitudes. The relevant sections were now revised according to the suggestion.

(Line 617-627)

FEP comparisons across conditions

"Despite the lower rate of tactile perception, FUS stimulation elicited distinct EEG features across all subjects. The acquired FEP shared similarity with SSEP features that were elicited by unilateral median nerve stimulation (e.g., a negative peak appearing with 80–100 ms latencies followed by a positive peak having ~200 ms latencies; **Fig. 3**) whereas the sham condition did not elicit any specific EEG peaks. However, these peaks appeared much later than the those of SSEP (the first negative peak at 51 ms and positive peak at 150 ms upon electrical stimulation). Furthermore, the FEP showed smaller EEG peak magnitudes. We surmise that these differences between FEP and SSEP stemmed from different mechanisms (SSEP reflects afferent brain activity responding to peripheral stimulation whereas FEP detects the neuronal activity caused by external brain stimulation). Despite these differences, these results suggest the involvement of overlapping neuronal pathways between the responses to stimulations."…

We believe that the manuscript has improved based on the valuable comments, and we hope that it is now acceptable for publication.
